# Cholesterol suppresses human iTreg differentiation and nTreg function through mitochondria-related mechanisms

**DOI:** 10.1186/s12967-023-03896-z

**Published:** 2023-03-27

**Authors:** Huanzhi Zhang, Ni Xia, Tingting Tang, Shaofang Nie, Lingfeng Zha, Min Zhang, Bingjie Lv, Yuzhi Lu, Jiao Jiao, Jingyong Li, Xiang Cheng

**Affiliations:** 1 Department of Cardiology, Union Hospital, Tongji Medical College, Huazhong University of Science and Technology, Wuhan, China; 2 Hubei Key Laboratory of Biological Targeted Therapy, Union Hospital, Tongji Medical College, Huazhong University of Science and Technology, Wuhan, China; 3 Hubei Provincial Engineering Research Center of Immunological Diagnosis and Therapy for Cardiovascular Diseases, Union Hospital, Tongji Medical College, Huazhong University of Science and Technology, 1277 Jiefang Avenue, Wuhan, 430022 China

**Keywords:** Atherosclerosis, Regulatory T cells, Cholesterol, Mitochondrial reactive oxygen species, mitoTEMPO

## Abstract

**Background:**

Both the crystalline and soluble forms of cholesterol increase macrophage secretion of interleukin 1β (IL-1β), aggravating the inflammatory response in atherosclerosis (AS). However, the link between cholesterol and regulatory T cells (Tregs) remains unclear. This study aimed to investigate the effect of cholesterol treatment on Tregs.

**Methods:**

Differentiation of induced Tregs (iTregs) was analyzed using flow cytometry. The expression of hypoxia-inducible factor-1a (HIF-1a) and its target genes was measured by western blotting and/or RT-qPCR. Two reporter jurkat cell lines were constructed by lentiviral transfection. Mitochondrial function and the structure of natural Tregs (nTregs) were determined by tetramethylrhodamine (TMRM) and mitoSOX staining, Seahorse assay, and electron microscopy. The immunoregulatory function of nTregs was determined by nTreg-macrophage co-culture assay and ELISA.

**Results:**

Cholesterol treatment suppressed iTreg differentiation and impaired nTreg function. Mechanistically, cholesterol induced the production of mitochondrial reactive oxygen species (mtROS) in naïve T cells, inhibiting the degradation of HIF-1α and unleashing its inhibitory effects on iTreg differentiation. Furthermore, cholesterol-induced mitochondrial oxidative damage impaired the immunosuppressive function of nTregs. Mixed lymphocyte reaction and nTreg-macrophage co-culture assays revealed that cholesterol treatment compromised the ability of nTregs to inhibit pro-inflammatory conventional T cell proliferation and promote the anti-inflammatory functions of macrophages. Finally, mitoTEMPO (MT), a specific mtROS scavenger, restored iTreg differentiation and protected nTreg from further deterioration.

**Conclusion:**

Our findings suggest that cholesterol may aggravate inflammation within AS plaques by acting on both iTregs and nTregs, and that MT may be a promising anti-atherogenic drug.

**Supplementary Information:**

The online version contains supplementary material available at 10.1186/s12967-023-03896-z.

## Background

Atherosclerosis (AS) is characterized by the accumulation of cholesterol-carrying low-density lipoprotein (LDL) in the artery wall, with atherosclerotic plaques having a high cholesterol level [1]. In 1910, the German chemist Adolf Windaus found that AS plaques from the human aorta contained 25-fold more cholesterol than did non-AS samples. Using targeted lipidomic analysis, human atherosclerotic plaques were confirmed to contain large amounts of cholesterol [2].

Although AS was previously believed to be characterized by the passive build-up of cholesterol in the vessel wall, it is now recognized as a chronic inflammatory condition [3]. Results of clinical trials and studies on genome-wide association, in vivo imaging and transgenic lineage-tracing mice have clarified that both innate and adaptive immune pathways contribute to AS [4]. As the first successful immunotherapy trial for cardiovascular disease (CVD) [5], the CANTOS trial reported that interleukin 1b (IL-1β), a pro-inflammatory cytokine produced by myeloid cells, was neutralized by the antibody canakinumab, decreasing CVD occurrence by 15%, thereby demonstrating that inflammation is a promising drug target for CVD treatment.

Recent studies have reported the link between cholesterol and inflammation within AS plaques. Duewell et al. demonstrated that crystalline cholesterol activated the NLRP3 inflammasome and promoted the subsequent release of IL-1β from macrophages, which aggravated inflammation in AS plaques [6]. Menegaut et al. reported that liquid cholesterol and its hydroxylated derivatives could also increase IL-1β secretion in human macrophages via the LXR-HIF1α axis [2]. These results suggest that cholesterol deposition in arteries may be a critical cause of inflammation.

Regulatory T cells (Tregs), a specific subpopulation of T cells, comprise 5–10% of all peripheral CD4^+^ T cells and are critical in the induction and maintenance of immune homeostasis and tolerance. Forkhead/winged helix transcription factor (Foxp3) expression is a hallmark of Tregs and essential for their maturation and function; a lack of Foxp3 can result in Treg cell functional defect. Tregs protect mice and humans from AS through various mechanisms, including the secretion of anti-inflammatory cytokines such as interleukin 10 (IL-10) and transforming growth factor-b (TGF-β); suppression of pro-inflammatory conventional T (Tconv) cell proliferation; and promotion of anti-inflammatory phenotype conversion in macrophages [7]. Based on their developmental origin, Treg cells are classified into two major groups as follows: natural Tregs (nTregs) and induced Tregs (iTregs). The former develops directly in the thymus, whereas the latter differentiates from conventional CD4^+^ T cells [8]. Recently, single-cell RNA sequencing confirmed that both nTregs and iTregs exist in atherosclerotic plaques [9].

Cholesterol exacerbates the inflammatory status of AS plaques by interacting with immune cells, such as macrophages. Although relationships between cholesterol and T cells have been reported recently [10–13], little is known about the effects of cholesterol on Tregs. In this research, we investigated how cholesterol influenced iTreg differentiation and nTreg function, and further studied the related mechanisms. Our findings suggest that the mitochondrial reactive oxygen species (mtROS) scavenger, mitoTEMPO (MT), may be a promising anti-atherogenic drug.

## Methods

### PBMCs isolation

Human peripheral blood mononuclear cells (PBMCs) were isolated by density-gradient centrifugation with blood buffy coats provided by Wuhan Blood Center. Briefly, buffy coats were diluted 1:1 with phosphate buffer saline (PBS) and carefully layered over the lymphocyte separation medium (Solarbio, China) in 50 ml tube. Tubes were then centrifuged at 800 g with no brake for 20 min at room temperature (RT). PBMCs layer was carefully aspirated and washed with PBS for 3 times for next use.

### iTreg differentiation

To investigate the differentiation of iTreg, CD4^+^ naïve T cells were purified using a commercially available isolation kit according to the manufacturer’s instructions (Miltenyi Biotec, Germany) and stimulated with plate-coated anti-CD3 antibody (1 mg/ml, Biolegend, USA) and soluble anti-CD28 antibody (1 mg/ml, Biolegend, USA) in X-VIVO15 medium (Lonza, Switzerland). Three days after plating, TGF-b (1 ng/ml, Biolegend, USA) and interleukin 2 (IL-2, 50 U/ml, Biolegend, USA) were added in the culture, and the cells were continued to cultivate for 2 days. According to different experimental purposes, reagents were added together with the polarization cytokines, including methyl-b-cyclodextrin-cholesterol (cholesterol, 10–20 mg/ml, Sigma, USA), dimethyloxalylglycine (DMOG, 100 mM, MCE, USA), PX-478 (10 mM, MCE, USA), MT (10 mM, Sigma, USA) and IL-1b (100 ng/ml, Biolegend, USA).

### Cell counting kit 8 (CCK8) assay

CD4^+^ naïve T cells cultivated in 96-well plates were treated with cholesterol (5–20 mg/ml, Sigma, USA) at day 0 or day 3 post plating and continued to cultivate to day 5. CCK8 (Vazyme Biotec, China) solution was added to each well, and the plates were incubated at 37 ℃ for an additional 2 h. Culture medium of each well was mixed thoroughly by pipetting, and the plates were centrifuged at 3000 rpm, RT for 10 min to pellet cells. 100 ml aliquots of supernatant were measured at 450 nm of absorbance using a microplate reader. Viability of the cells in experimental groups was normalized to that of the cells in control group.

### Cell apoptosis assay

Cell apoptosis staining was performed using a commercially available apoptosis assay kit according to the manufacturers’ instructions (Thermo Fisher Scientific, USA). Briefly, cells were stained with Annexin V for 10 min at RT, and centrifuged and re-suspended in PI-containing binding buffer. The percentage of live cells (Annexin V^−^PI^−^), early (Annexin V^+^PI^−^) and late (Annexin V^+^PI^+^) apoptotic cells were determined by flow cytometry analysis.

### Flow cytometry analysis

For intracellular transcription factor Foxp3 staining, cells were fixed in the fixation/permeabilization buffer solution (Thermo Fisher Scientific, USA) for 30 min at 4 ℃, and then centrifuged and re-suspended in 100 ml permeabilization buffer (Thermo Fisher Scientific, USA). PE-Foxp3 antibody (Biolegend, USA) was added to stain the cells for 30 min at 4 ℃, and cells were washed with permeabilization buffer for 2 times.

For mitoSOX staining, cells were incubated within 5 mM mitoSOX red dye (Thermo Fisher Scientific, USA) solution for 10 min at 37 ℃ in cell culture incubator. For tetramethylrhodamine (TMRM) staining, cells were incubated within 0.1 mM TMRM (Invitrogen, USA) solution for 30 min at 37 ℃ in cell culture incubator.

### Western blotting

Cells were lysed in radioimmunoprecipitation assay (RIPA) buffer supplemented with protease inhibitor. The protein concentration was determined using a BCA protein assay kit (Thermo Fisher Scientific, USA). Equal amounts of protein were subjected to electrophoresis on a 4–20% FuturePAGE gel (ACE Biotec, China) and then transferred to polyvinylidene fluoride (PVDF) membranes (Merck Millipore, Germany). The membranes were blocked with 7% nonfat milk and incubated with primary antibodies against HIF-1a (1:250, R&D, USA) and b-actin (1:1000, Abcam, UK) overnight at 4 °C. The PVDF membranes were then incubated with horseradish peroxidase (HRP)-conjugated secondary antibody at RT for 1 h. The protein bands on the PVDF membranes were observed using an enhanced chemiluminescence kit (Biosharp, China) and quantified using ImageJ.

### nTreg isolation, expansion and treatment

nTreg cells were isolated using the EasySep^™^ Human CD4^+^CD127^low^CD25^+^ Regulatory T Cell Isolation Kit (Stemcell, Canada) according to the manufacturer’s instructions. The sorted nTregs were plated at 10^5^ cells per well in 96-well plates and expanded with the Dynabeads human Treg expander (Thermo Fisher Scientific, USA) at a 1:1 bead/cell ratio in IL-2 (1000 U/ml, Biolegend, USA) supplemented X-VIVO15 medium (Lonza, Switzerland) (Additional file 2: Figure S1). Fresh medium was replenished when the culture became yellow. At days 5–8, nTreg cells were transferred into a 24-well plate. On day 9, cells were re-stimulated with fresh beads at a 1:1 bead/cell ratio. On day 14, after removal of the Dynabeads with a magnet, nTregs were harvested and replated in fresh X-VIVO15 medium containing 100 U/ml IL-2 to rest for 24 h. During the rest period, cholesterol (10–20 mg/ml, Sigma, USA) was added to the culture, alone or combined with MT (50 mM, Sigma, USA), to stimulate nTregs for 16 h.

### Mixed lymphocyte reaction (MLR)

Freshly isolated CD4^+^CD25^−^ conventional T (Tconv) cells were labeled with CellTrace Violet (CTV, Thermo Fisher Scientific, USA) and co-cultured with nTregs at a 4:1 ratio for 4 days. Anti-CD3 antibody (1 mg/ml) was coated onto the bottom of the plate, and soluble anti-CD28 antibody (1 mg/ml) was added in the culture medium. Mean fluorescence intensity (MFI) of CTV at day 4 was measured by flow cytometry.

### Macrophage-nTreg co-culture

Human monocytic THP-1 cells were maintained in RPMI 1640 culture medium (Hyclone, USA) supplemented with 10% fetal bovine serum (FBS, Gibco, USA). THP-1 monocytes were plated in 48-well plates at 1.5 × 10^5^ cells per well and differentiated into macrophages by incubation with 150 nM PMA (Sigma, USA) overnight. nTregs were added to the culture and incubated with macrophages for 48 h at a 4:1 macrophage/nTreg ratio in the presence of anti-CD3 antibody (50 ng/ml, Biolegend, USA). After that, supernatant containing nTregs was aspirated and discarded, and the plates were washed 3 times with PBS for next use.

### In vitro efferocytosis assay

CTV-labeled THP-1 monocytes were differentiated into macrophages and co-cultured with nTregs as described above. Jurkat cells labeled with CFSE (Thermo Fisher Scientific, USA) were rendered apoptotic by incubating with the anti-Fas antibody (200 ng/ml, Biolegend, USA) for 2 h. Then macrophages were incubated with Jurkat cells at a 1:2 ratio for 2 h. After vigorously washing with PBS, the cells were harvested using the EDTA-trypsin solution (Gibco, USA). In vitro efferocytosis (CTV^+^CFSE^+^) was determined by flow cytometry analysis.

### In vitro foam cell formation assay

THP-1 monocytes were differentiated into macrophages and co-cultured with nTregs as described above. Then macrophages were incubated with 30 mg/ml Dil-labeled oxidized low-density lipoprotein (Dil-oxLDL, Yiyuan Biotec, China) for 4 h. Cells were harvested and MFI of Dil was determined with flow cytometry analysis.

### Real-time quantitative polymerase chain reaction (RT-qPCR)

Total RNA was extracted with TRIzol isolation reagent (Vazyme Biotec, China) and reverse transcribed into cDNA using the HiScript RT supermix (Vazyme Biotec, China) according to the manufacturer’s instructions. RT-qPCR were prepared with sequence-specific primers (Additional file 1: Table S1) and ChamQ SYBR qPCR Master Mix (Vazyme Biotec, China) in a 10 μl volume. RT-qPCR was performed using a CFX96 Real-Time PCR Detection System (Bio-Rad, USA) and each reaction was performed in duplicate. Data from each sample were standardized with GAPDH or 18S using the 2^−ΔΔCT^ method.

### Electron microscopy

nTreg cells were treated with cholesterol alone or combined with MT for 16 h as described above. For electron microscopy, nTregs were sequentially fixed with 2.5% glutaraldehyde and 1% osmium tetroxide on ice. Then cells were dehydrated in a graded ethanol series and embedded in resin mixture. Ultrathin cell slices of different groups were obtained using a ultramicrotome and subsequently mounted onto copper grids and stained with uranyl acetate and lead citrate. Mitochondrial morphology was examined with a FEI Tecnai G2 20 TWIN transmission electron microscope.

### Measurement of OCR (oxygen consumption rate) and glycoPER (glycolysis proton efflux rate) by Seahorse analysis

The Seahorse sensor probe plate was hydrated with XF calibrant 10 h before the test. The 24-well cell culture plates were coated with Poly-l-lysine (50 mg/ml, Solarbio, China) according to manufacturer’s instructions. 200,000 cells were plated in 200 ml XF Seahorse medium (pH 7.4, Agilent, USA) supplemented with 2 mM glutamine, 10 mM glucose and 1 mM sodium pyruvate. Plates were centrifuged at 200 g without break and cells were then incubated for 30 min at 37 ℃ without CO_2_. Afterwards, 300 ml of the same XF medium was added to each well. OCR was measured at basal conditions and after sequential stimulation of the cells by 1 mM Oligomycin (Oligo), 1 mM carbonyl cyanide p-trifluoromethoxy phenylhydrazone (FCCP) and 0.5 mM Rotenone/Antimycin (Rot/AA) (all included in the Mitostress kit, Agilent, USA). After the same pre-treatment, glycoPER was measured under basal conditions and with the sequential addition of 0.5 μM Rot/AA and 50 mM 2-deoxy-D-glucose (2-DG) (all included in the Glycolytic rate kit, Agilent, USA).

### Enzyme-linked immunosorbent assay (ELISA)

To assay interleukin 4 (IL-4), IL-10, interleukin 13 (IL-13) and TGF-b, nTregs were plated in 48-well plates and stimulated with 50 mg/ml anti-CD3 antibody (Biolegend, USA) for 48 h. After that, conditioned culture medium was collected and pelleted in order to get rid of cells. 50 ml aliquots of the medium samples were assayed using the commercially available ELISA kits (Bioswamp, China) according to the manufacturer’s instructions.

To assay 8-hydroxydeoxyguanosine (8-OHdG), nTregs were pelleted and ultrasonic lysed within PBS. The cellular lysates were centrifuged at 12,000 rpm for 10 min to remove cell debris. 40 ml aliquots of cellular lysate were assayed using a commercially available ELISA kit (Bioswamp, China) according to the manufacturer’s instructions.

### Lentiviral transfection

A lentivirus carrying the oxygen-dependent degradation domain-luciferase (ODD-Luc) fusion protein comprising HIF-1α (amino acid 530–652, a unique ODD) fused to the N-terminus of firefly luciferase was designed and produced by DesignGene Biotec, Co., Ltd. (Shanghai, China) (Additional file 1: Table S2). Jurkat cells were seeded at 10^5^ cells per well in 24-well plates and maintained in RPMI 1640 culture medium (Hyclone, USA) supplemented with 10% FBS (Gibco, USA). Lentivirus carrying ODD-Luc was added to the medium at a multiplicity of infection of 50 to transfect Jurkat cells for 3 days. Fresh culture medium was replenished, and the cells were then cultivated under standard culture conditions (37 ℃, 5% CO_2_) for an additional 2 days. Finally, 5 µg/ml puromycin (Biosharp, China) was added to the culture to select stably transfected cells for subsequent use.

Lentiviruses carrying hypoxia-responsive elements-green fluorescent protein (HREs-GFP) were designed and produced by DesignGene Biotec (Additional file 1: Table S2). Similarly, the HREs-GFP reporter Jurkat cell line was generated by lentivirus transfection and subsequent puromycin selection, and stably transfected cells were prepared for subsequent use.

### Dual luciferase reporter assay

Jurkat cells were seeded at 2.5 × 10^6^ cells per well in 6-well plates and stimulated with different reagents, including cholesterol (20µg/ml, Sigma, USA) alone and in combination with MT (10 µM, MCE, USA), DMOG (100 µM, MCE, USA), and MG-132 (10 µM, MCE, USA). The cells were then pelleted and lysed in a cell lysis buffer. After centrifugation at 12000 g, RT for 2 min to remove cell debris, 20 µl aliquots of the cellular lysate were assayed using a commercially available dual luciferase reporter assay kit (Vazyme Biotec, China) according to the manufacturer’s instructions. The activities of firefly and Renilla luciferase were evaluated using a full-wavelength microplate reader. Firefly luciferase activity was normalized to Renilla luciferase activity and expressed relative to that of the control group.

### HREs-GFP reporter assay

HREs reporter Jurkat cells were seeded at 0.5 × 10^6^ cells per well in 12-well plates and stimulated with cholesterol (20µg/ml, Sigma, USA) alone or in combination with PX-478 (10 µM, MCE, USA) or MT (10 µM, MCE, USA) for 48 h. Cells were harvested and fixed, and the MFI of GFP was measured by flow cytometry.

### Statistics

Data were presented as the mean ± standard error of the mean (SEM). The significance of differences between two groups was evaluated using unpaired Student’s t test, and the differences for multiple groups were analyzed using one-way ANOVA followed by Tukey’s tests. A *P* < 0.05 was considered statistically significant.

## Results

### Cholesterol inhibits iTreg differentiation in vitro

**Fig. 1:**
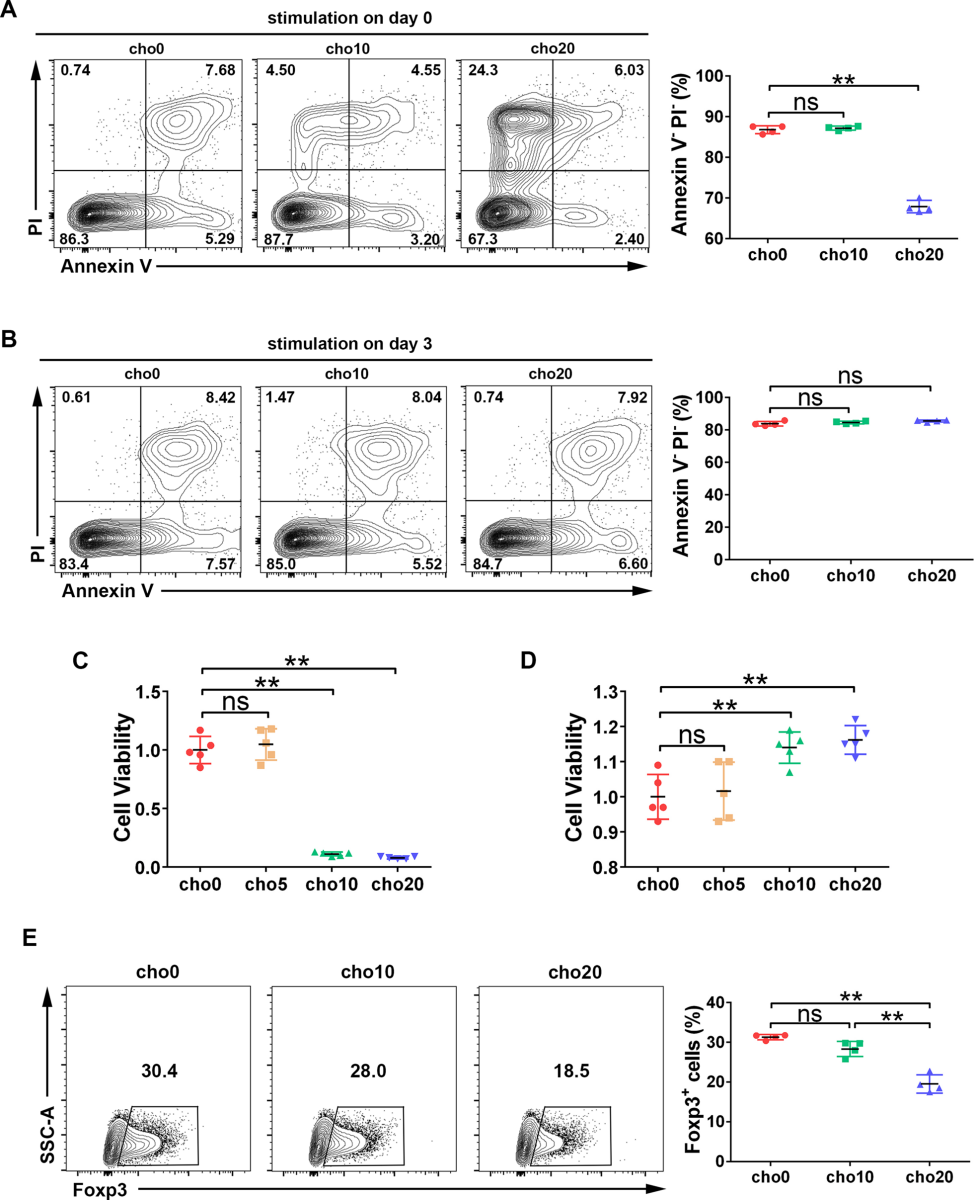
Cholesterol treatment at day 3 post plating inhibits iTreg differentiation. Naïve T cells were treated with cholesterol at the day of plating (A, C) or 3 days after plating (B, D). **A, B** Cellular apoptosis was measured by Annexin V/PI staining. n = 4 per group. **C, D** Cell viability was determined by CCK8 assay. n = 5 per group. **E** Naïve T cells were stimulated with cholesterol and iTreg polarization cytokines at day 3 post-plating and continued to cultivate for 2 days. Frequency of Foxp3.^+^ cells was analyzed by flow cytometry. n = 4 per group. Data are expressed as the mean ± SEM. *P < 0.05, **P < 0.01

To determine how cholesterol affects iTreg differentiation, we cultured CD4^+^ naïve T cells under iTreg polarization conditions in the presence of 0–20 mg/ml of cholesterol (indicated as cho0–20). First, we found that adding cholesterol at different times after plating yielded different results. Freshly isolated naïve T cells were sensitive to cholesterol treatment; stimulation on day zero impaired cell viability, manifested as low cell proliferation and high cell death (Fig. 1A, C). However, when the exponential growth period was reached (approximately 3 days after plating), no cell damage resulted from the addition of cholesterol up to 20 µg/ml, as evidenced by Annexin V/PI apoptosis staining (Fig. 1B). CCK8 assays showed that cholesterol addition on day 3 mildly but significantly improved cell viability; this may be due to the increase in cell proliferation, as described previously (Fig. 1D) [14, 15]. Furthermore, the rapid cell proliferation consumed the additional cholesterol, offsetting its damaging effect [16]. Based on the above findings, we chose to add cholesterol and iTreg polarization cytokines, including TGF-β and IL-2, on the 3rd day after cell plating, and the cells were then cultured for 2 days. Cholesterol suppressed iTreg differentiation in a dose-dependent manner (Fig. 1E).

### Cholesterol depresses iTreg differentiation by inducing HIF-1α expression

**Fig. 2:**
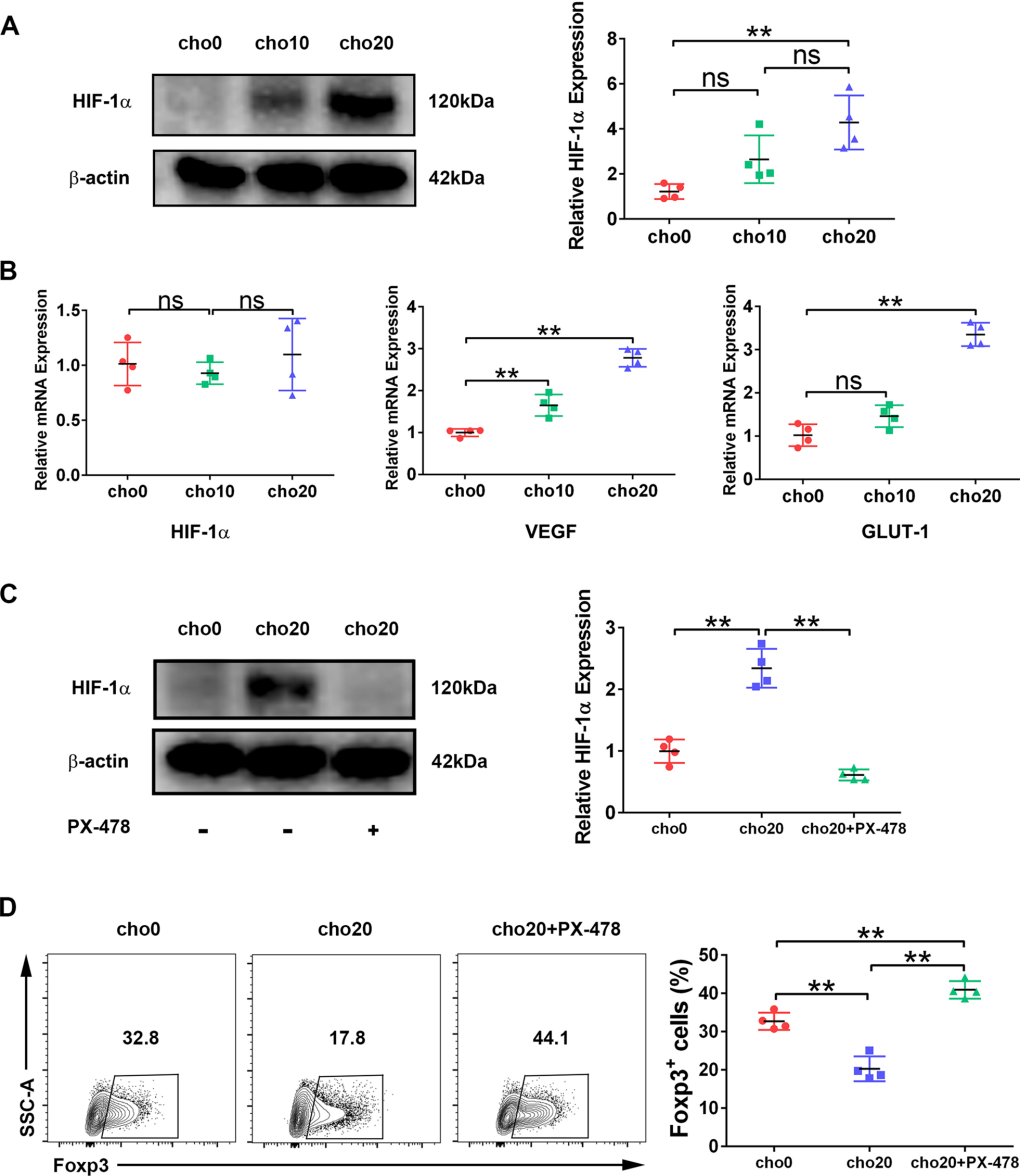
Cholesterol attenuates iTreg differentiation by inducing HIF-1a expression. **A** Protein levels of HIF-1a were analyzed by western blotting at day 2 of polarization with different doses of cholesterol added in the culture. Expression of b-actin was used as a loading control. n = 4 per group. **B** Relative mRNA levels of HIF-1a, VEGF and GLUT-1 were measured by RT-qPCR. n = 4 per group. **C** Protein levels of HIF-1a were analyzed at day 2 of polarization in the presence or absence of PX-478. n = 4 per group. **D** The percentage of Foxp3.^+^ cells at day 2 of polarization was measured with flow cytometry. n = 4 per group. Data are expressed as the mean ± SEM. *P < 0.05, **P < 0.01

We then sought to determine the mechanism by which cholesterol inhibits iTreg differentiation. HIF-1α plays an important role in suppressing iTreg differentiation in both humans and mice by binding Foxp3 and targeting it for proteasomal degradation [17]. Cholesterol can stabilize HIF-1α expression under normoxia in hepatocytes [18, 19]; we therefore investigated the effects of cholesterol on HIF-1α expression in iTregs. A higher HIF-1α signal was detected by western blotting on day 2 of polarization when cholesterol was added to the cultures (Fig. 2A). Meanwhile, the mRNA levels of HIF-1α target genes, including *VEGF* and *GLUT-1*, were also upregulated, although *HIF-1a* transcription did not change significantly (Fig. 2B). However, inhibition of HIF-1α using an inhibitor, PX-478, abrogated the suppressive effect of cholesterol and restored the percentage of Foxp3^+^ cells to an even higher level than that in the control group (Fig. 2C, D). IL-1b was reported to inhibit iTreg polarization by upregulating HIF-1a expression [20], so we studied whether cholesterol could play a synergistic role with IL-1b in dampening iTreg differentiation. As shown in Additional file 2: Figure S2, when cholesterol was added together with IL-1b, Foxp3^+^ cell percentage was further decreased.

### Induction of HIF-1α expression relies on mtROS production triggered by cholesterol

Reactive oxygen species (ROS) are strong inducers of HIF-1α expression. mtROS serve as intracellular HIF-1α -stabilizing signals in various cells, including monocytes, CD4^+^ lymphocytes, and hepatocytes [18, 21, 22]. Baseline mitochondrial cholesterol levels are low and mitochondrial cholesterol accumulation can lead to increased production of mtROS [23]. Therefore, we investigated whether cholesterol was also associated with the mtROS-HIF-1α mechanism during iTreg differentiation.

**Fig. 3:**
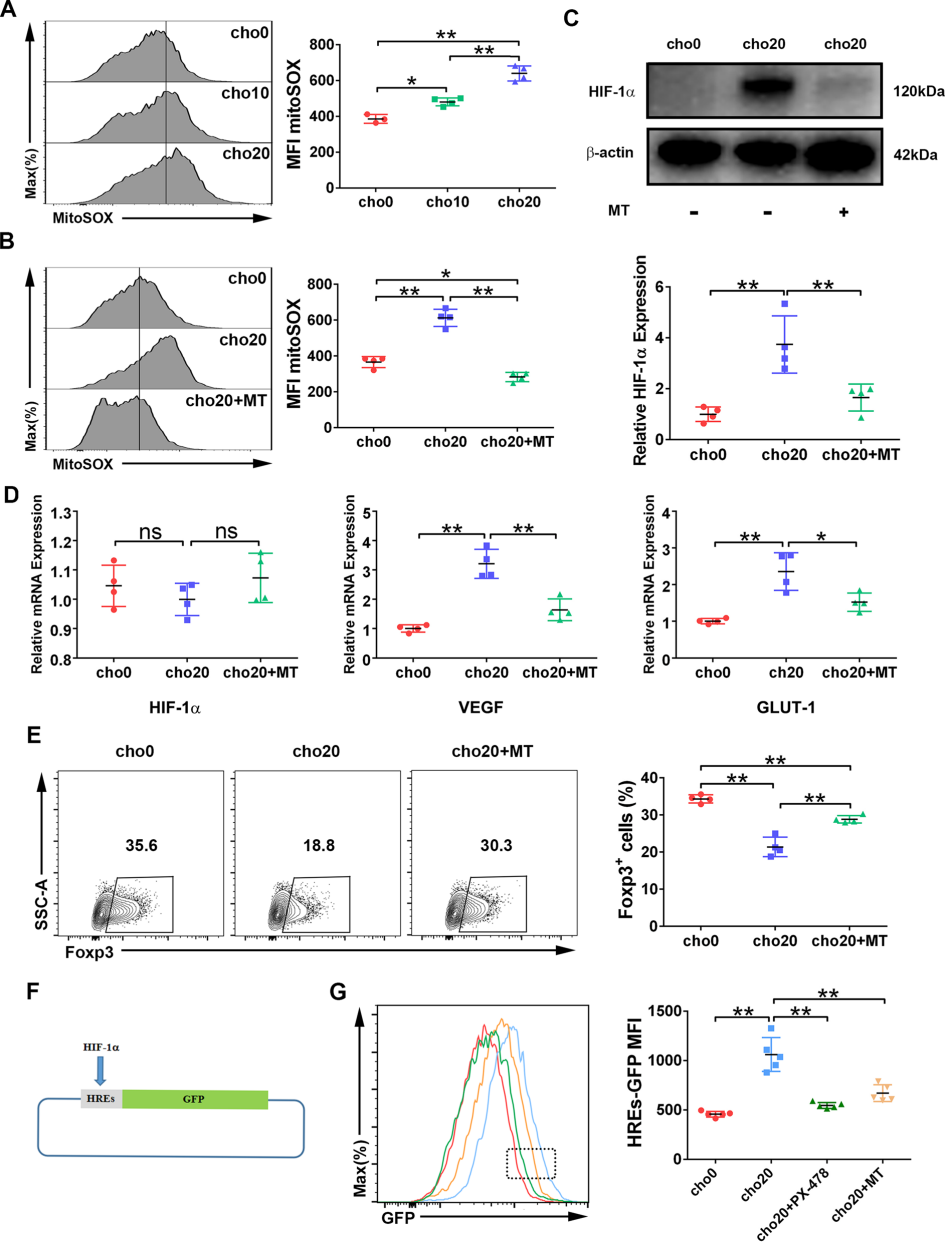
HIF-1a expression is induced by mtROS. Naïve T cells were polarized for 2 days with cholesterol added alone or combined with MT. **A-B** mtROS were analyzed with mitoSOX staining. n = 3–4 per group. **C** Protein levels of HIF-1a were analyzed by western blotting. n = 4 per group. **D** Relative mRNA levels of *HIF-1a*, *VEGF* and *GLUT-1* were measured by RT-qPCR. n = 4 per group. **E** The percentage of Foxp3.^+^ cells at day 2 was measured by flow cytometry. n = 3–4 per group. **F** HREs-GFP reporter Jurkat cell line was constructed. **G** HREs-GFP reporter Jurkat cells were stimulated with the indicated reagents for 2 days, and MFI of GFP was measured by flow cytometry. n = 5 per group. Data are expressed as mean ± SEM. *P < 0.05, **P < 0.01

First, we evaluated mtROS production using the probe mitoSOX and found that cholesterol stimulation significantly increased mtROS in iTregs (Fig. 3A). However, the elevation of mtROS levels was reduced by MT, a mitochondria-specific superoxide scavenger (Fig. 3B). We then investigated whether MT reduced HIF-1α expression, and found that it visibly abrogated the expression of HIF-1α (Fig. 3C). The mRNA expression of both *VEGF* and *GLUT-1* was also downregulated (Fig. 3D). Meanwhile, MT reversed the cholesterol-induced reduction in the percentage of Foxp3^+^ cells (Fig. 3E).

Finally, we constructed a HREs-GFP reporter Jurkat cell line, in which the expression of the reporter (GFP) was under the control of HREs, the DNA regulatory sequences present in the promoter or enhancer regions of HIF-1α target genes (Fig. 3F) [22]. Cholesterol stimulation induced GFP expression in reporter Jurkat cells, however both PX-478 and MT abrogated this effect (Fig. 3G); these results are consistent with those derived from iTreg cells.

### mtROS inhibit HIF-1α protein degradation

**Fig. 4:**
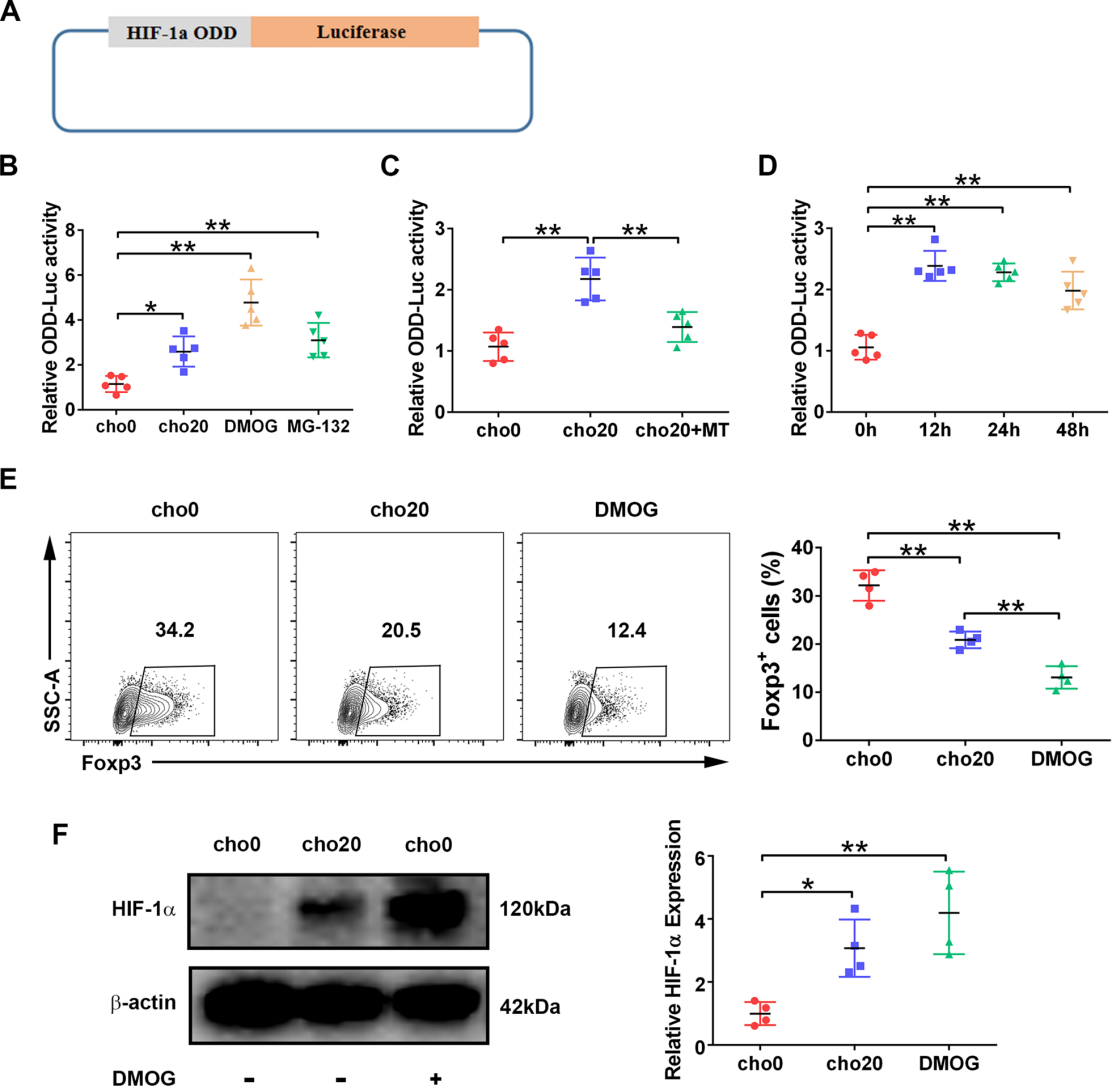
mtROS inhibit HIF-1a degradation. **A** ODD-Luc reporter Jurkat cell line was constructed. **B** ODD-Luc reporter Jurkat cells were treated with cholesterol, DMOG, or MG-132 for 48 h, and luciferase activity was measured. n = 5 per group. **C** ODD-Luc reporter Jurkat cells were treated with cholesterol alone or combined with MT for 48 h, and luciferase activity was measured. n = 5 per group. **D** ODD-Luc reporter Jurkat cells were stimulated with 20 mg/ml of cholesterol for 12–48 h, and luciferase activity was measured. n = 5 per group. **E** Naïve T cells were polarized for 2 days in the presence or absence DMOG. The percentage of Foxp3.^+^ cells was measured with flow cytometry. n = 4 per group. **F** Protein levels of HIF-1a were analyzed by western blotting at day 2 of polarization. n = 4 per group. Data are expressed as the mean ± SEM. *P < 0.05, **P < 0.01

Under normoxia, HIF-1α is hydroxylated by prolyl hydroxylase-domain enzymes (PHDs) and targeted for proteasomal degradation [24]. PHDs are sensitive to ROS oxidation, which can lead to their inactivation and subsequent HIF-1a accumulation [25]. To test whether there is a similar mechanism in cholesterol-treated T cells, we constructed an ODD-Luc reporter Jurkat cell line that carries a fusion protein consisting of HIF-1α ODD fused to the N-terminus of firefly luciferase (Fig. 4A). This chimeric protein behaved similar to HIF-1α in living cells, and its luciferase activity was directly proportional to the inhibition of PHDs in HIF-1α ODD [26].

Incubation of reporter Jurkat cells with cholesterol led to increased luciferase activity, and treatment with the PHD inhibitor DMOG or proteasome inhibitor MG-132 generated a similar result (Fig. 4B), suggesting that cholesterol may increase HIF-1α levels in T cells by inhibiting its degradation. Furthermore, by scavenging mtROS, MT significantly reduced cholesterol-induced luciferase activity, indicating that mtROS mediated the HIF-1α stabilizing effect of cholesterol (Fig. 4C). Moreover, by analyzing luciferase activity, we investigated HIF-1α levels at different time points after cholesterol stimulation, and found that HIF-1α began to accumulate as early as 12 h after stimulation with cholesterol, and the effects lasted for at least 48 h (Fig. 4D).

Finally, we studied the effect of DMOG on iTreg polarization. DMOG treatment markedly abrogated iTreg differentiation (Fig. 4E), and up-regulated HIF-1α protein levels were also observed (Fig. 4F).

### Cholesterol compromises nTreg immunosuppressive activity by inducing mitochondrial oxidative stress

**Fig. 5:**
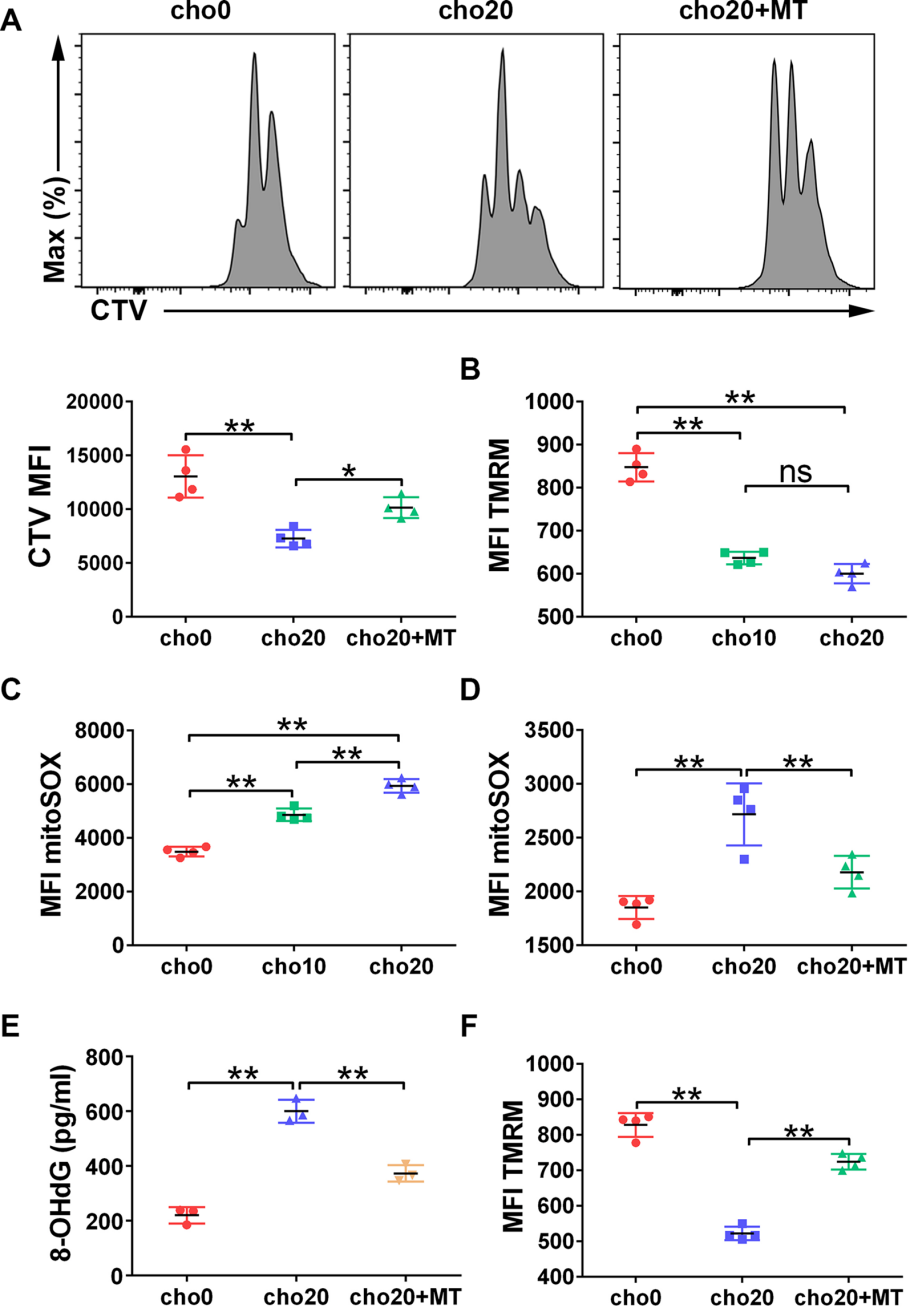
Cholesterol compromises nTreg cellular and mitochondrial function. In vitro expanded nTregs were treated with cholesterol alone or in combination with MT for 16 h. **A** nTregs were co-cultured with CTV labeled Tconv cells for 4 days. MFI of CTV was measured by flow cytometry. n = 4 per group. **B**, **E** MMP of nTregs was determined by TMRM staining. **C, D** mtROS were measured by mitoSOX staining. n = 4 per group. **F** Oxidative damage of nTregs was evaluated by 8-OHdG ELISA analysis. n = 3 per group. Data are expressed as the mean ± SEM. *P < 0.05, **P < 0.01

Next, we sought to investigate the effects of cholesterol on nTregs. In vitro expanded nTregs were plated to rest for 24 h. During this process, cholesterol was added to stimulate nTregs for 16 h. First, we used the MLR assay to assess the immunosuppressive activity of nTregs. Tconv cells in the cho20 group had a higher degree of CTV dilution than did the control group (Fig. 5A), indicating that the normal suppressive activity of nTregs was compromised by cholesterol treatment.

The suppressive function of nTregs was highly dependent on mitochondrial wellness [27]. However, intracellular accumulation of cholesterol reportedly led to mitochondrial dysfunction, which is always associated with depolarization of the mitochondrial membrane potential (MMP). Thus, we assessed the MMP of cholesterol-treated nTregs using a specific probe TMRM. Cholesterol treatment considerably diminished TMRM staining compared with the control group (Fig. 5B), indicating that cholesterol impairs mitochondrial function in nTregs.

A mtROS burst due to the mitochondrial dysfunction leads to cellular oxidative stress and further deterioration of mitochondrial fitness [28–30]. Our results showed that cholesterol treatment increased mtROS production in nTregs (Fig. 5C). Therefore, we investigated whether it can protect the mitochondrial and cellular function of nTregs by targeting mtROS. The mtROS scavenger MT was added into the culture while stimulating nTregs with cholesterol. We found that MT supplementation significantly reduced mtROS production (Fig. 5D), as well as resulting in an attenuated level of cellular oxidative stress indicated by 8-OHdG expression (Fig. 5E). MMP depolarization was also reversed (Fig. 5F). Moreover, as revealed by the MLR assay, MT treatment partially, but significantly, restored the immunosuppressive function of nTregs (Fig. 5A).

### Cholesterol impairs nTreg mitochondrial function and structure

**Fig. 6:**
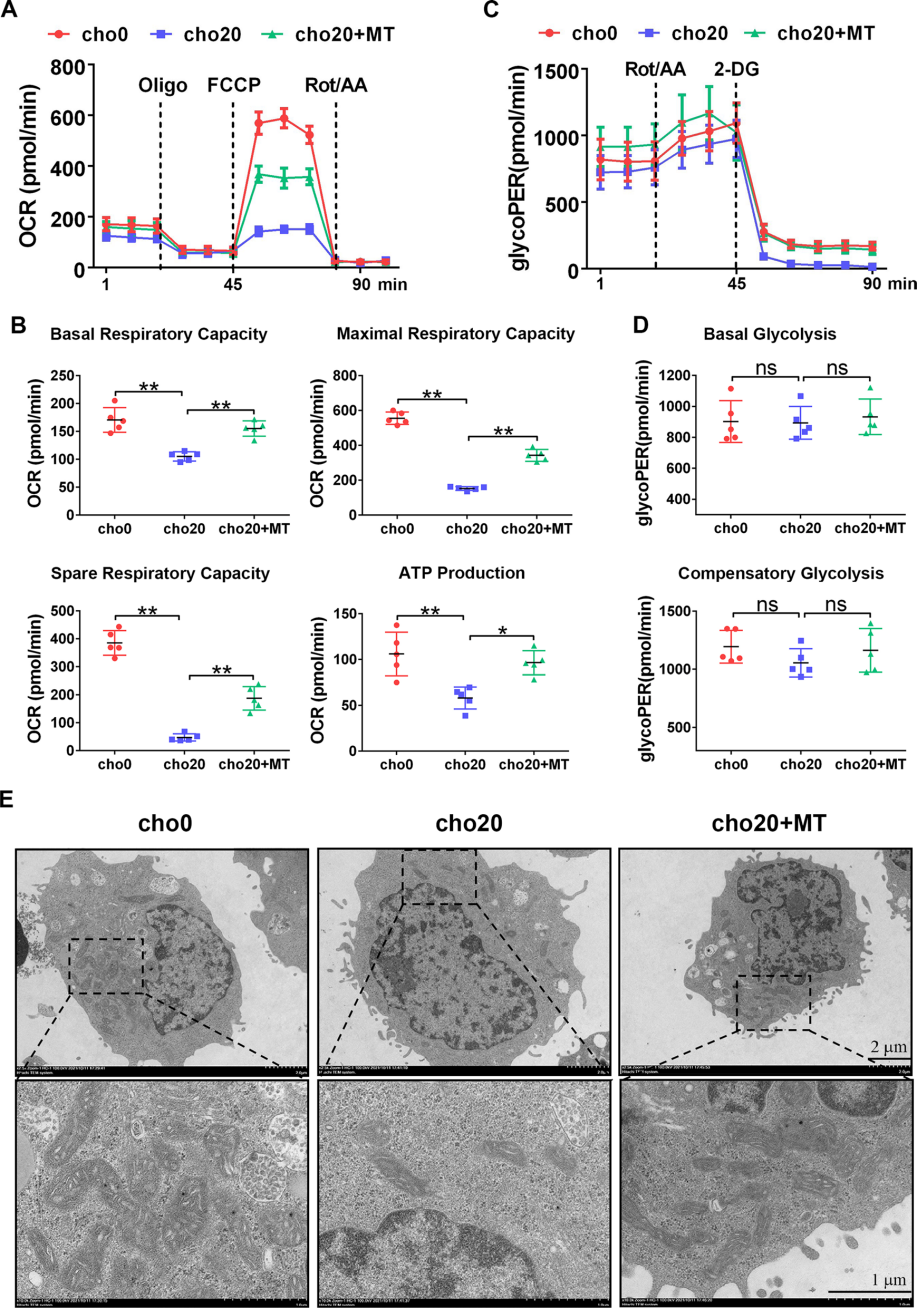
MT alleviates nTreg mitochondria functional and structural damage induced by cholesterol. In vitro expanded nTregs were treated with cholesterol alone or in combination with MT for 16 h. **A, B** OCR of nTregs was measured by Seahorse assay. n = 5 per group. **C, D** The glycoPER of nTregs was measured by Seahorse assay. n = 5 per group. **E** The mitochondrial number and structure of nTregs were examined with transmission electron microscopy. Data are expressed as the mean ± SEM. *P < 0.05, **P < 0.01

To directly assess the mitochondrial function, we measured the OCR of nTregs using the Seahorse assay. We observed that cho20-treated nTregs had a lower basal oxygen consumption rate and lower ATP production than did non-treated cells. Diminished spare and maximal respiratory capacities were also observed. However, when MT was added, nTreg mitochondrial respiration data were all restored (Fig. 6A, B). The glycoPER was also measured to assess the glycolytic capacities of nTregs. We found that both cholesterol and MT treatment did not affect the rates of basal or compensatory glycolysis in nTregs (Fig. 6C, D). Finally, we studied the effects of cholesterol and MT on the structure of nTreg mitochondria using the transmission electron microscopy. As shown in Fig. 6E, MT treatment reversed the formation of mitochondrial structural lesions induced by cholesterol, including reversal of the disruption to cristae organization and membrane integrity and of mitochondrial fragmentation. Mitochondria numbers in nTregs were also restored (Additional file 2: Figure S3) [31]. These results corroborate that cholesterol treatment impairs nTreg mitochondrial function and structure, which can be reversed by MT.

### Cholesterol depresses nTregs’ ability to regulate macrophage phenotype and function

Macrophages play a central role in the occurrence, development, and regression of AS. nTregs ameliorate AS, mainly by licensing the pro-resolving functions of macrophages and preventing macrophage-derived foam cell formation [5]. Using a widely used Treg-macrophage co-culture model simulating in vivo conditions, we studied whether cholesterol treatment could alter the ability of nTregs to regulate macrophage phenotype and function.

**Fig. 7:**
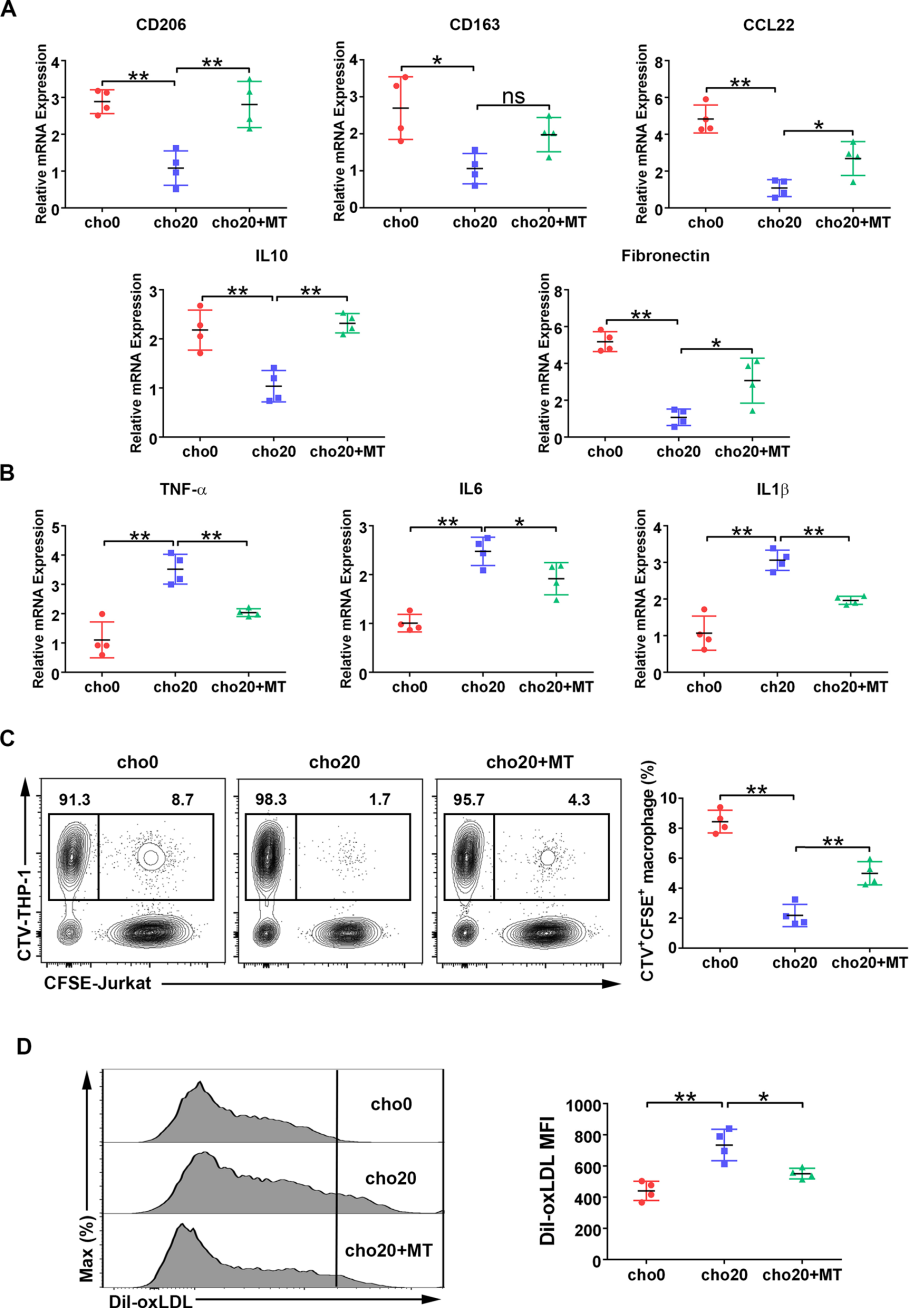
Cholesterol impairs nTregs’ immunoregulatory activity on macrophages. In vitro expanded nTregs were treated with cholesterol alone or combined with MT for 16 h and co-cultured with THP-1 macrophages for 2 days. **A, B** Relative mRNA levels of M2 (A) and M1 (B) maker genes were measured by RT-qPCR. n = 4 per group. **C** CTV-labeled macrophages were co-cultured with CFSE-labeled apoptotic Jurkat cells for 2 h. Phagocytosis of Jurkat by macrophages (gated on CTV^+^CFSE.^+^) was measured by flow cytometry. n = 4 per group. **D** Macrophages were incubated with Dil-oxLDL for 4 h, and their MFI was analyzed by flow cytometry. n = 4 per group. Data are expressed as the mean ± SEM. *P < 0.05, **P < 0.01

Using RT-qPCR, we first investigated the nTreg-induced polarization of macrophages [32]. We found that cholesterol-treated nTregs had a reduced ability to up-regulate typical M2-relevant markers and down-regulate M1-relevant markers of macrophages (Fig. 7A, B). Phagocytosis of apoptotic cells, known as efferocytosis, is a key process in the resolution of inflammation, and prevents apoptotic cells from becoming necrotic and pro-inflammatory. Tregs promoted macrophage efferocytosis and the resolution of multiple inflammatory diseases, including AS [9, 33]. Using an in vitro efferocytosis assay, we confirmed that cholesterol treatment decreased the ability of nTregs to promote the clearance of apoptotic Jurkat cells by THP-1 macrophages (Fig. 7C). Macrophage-derived foam cell formation is a hallmark of AS progression, which promotes the inflammatory rupture of AS plaques. Treg cells could inhibit foam cell formation by decreasing oxLDL uptake by macrophages [34]. However, we found that cholesterol stimulation compromised the ability of nTregs to inhibit foam cell formation (Fig. 7D). Next, we investigated whether MT could improve the immunoregulatory function of cholesterol-treated nTregs. As shown in Fig. 7A–D, MT treatment not only promoted nTreg-induced macrophage alternative (M2) activation, but also improved the phagocytic function of macrophages. Meanwhile, compared with the cho20 group, MT treatment decreased oxLDL uptake by macrophages. These in vitro results suggest that cholesterol-treated nTregs may have an impaired ability to ameliorate AS; nevertheless, MT could play a protective role.

**Fig. 8:**
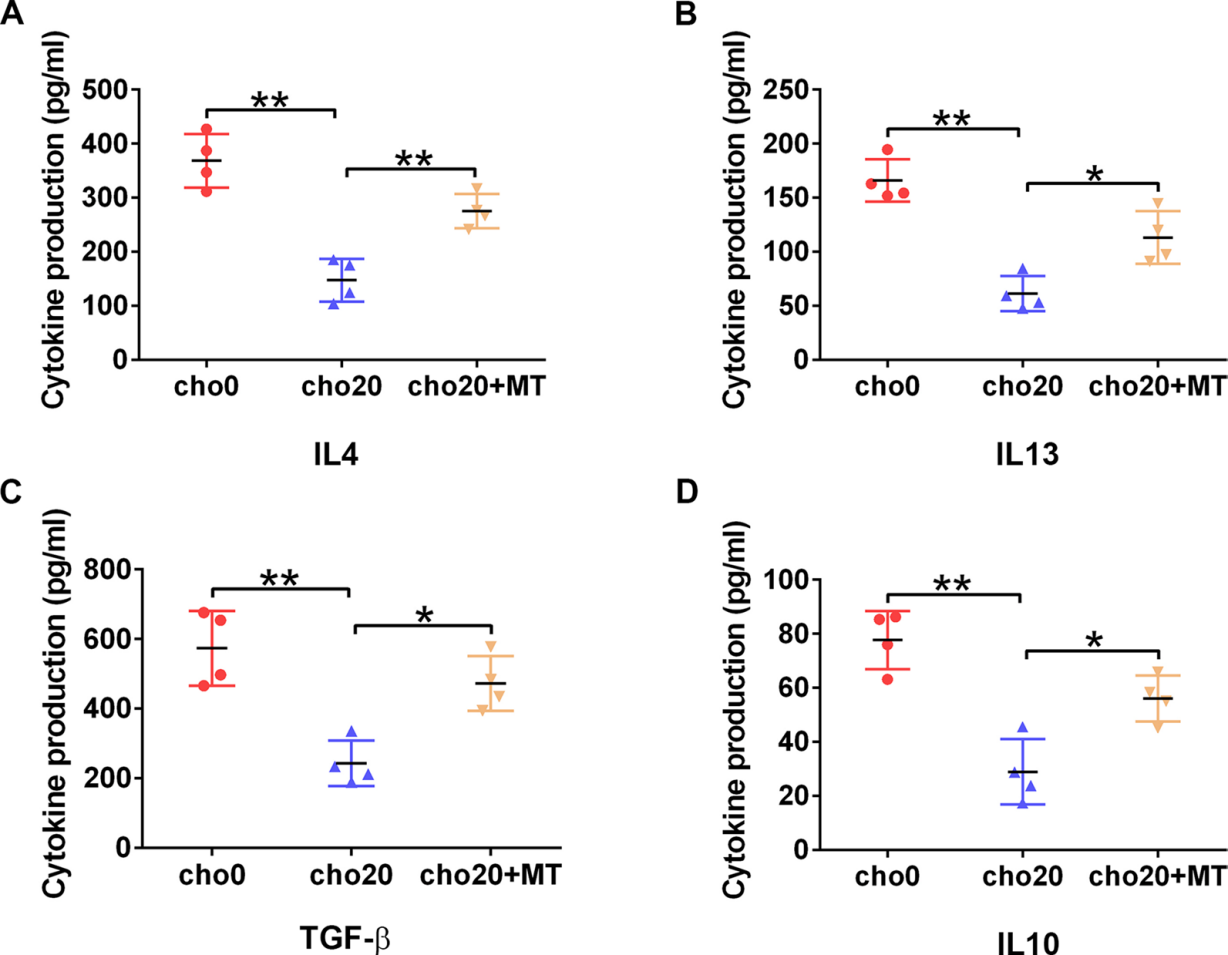
MT restores the cytokine secretion of cholesterol-treated nTregs. **A-D** nTreg cells were stimulated with cholesterol alone or in combination with MT for 16 h, and re-stimulated with anti-CD3 antibody for 2 days. The concentrations of IL-4, IL-10, IL-13 and TGF-b in the supernatant were measured by ELISA. n = 4 per group. Data are expressed as the mean ± SEM. *P < 0.05, **P < 0.01

Multiple cytokines secreted by nTregs, especially IL-4, IL-10, IL-13 and TGF-β, are involved in the functional and phenotypic shaping of macrophages, as revealed by Treg-macrophage co-culture experiments [32–34]. Our results indicated that cholesterol treatment considerably decreased the concentration of these four cytokines in the nTreg culture supernatant. However, after MT addition, they were restored to varying degrees (Fig. 8A–D). These results may partially account for the functional changes observed in nTregs induced by cholesterol and MT.

## Discussion

Tregs participate in the protection and repair of AS, which is characterized by the accumulation of cholesterol in the artery wall. However, it is not clear how cholesterol affects the phenotype and function of Tregs in AS. In this study, we found that cholesterol induced HIF-1α stabilization in naïve T cells and inhibited their differentiation into iTregs. Furthermore, cholesterol triggered nTregs mitochondrial oxidative damage, leading to the impairment of their immunosuppressive function.

HIF-1α, a key metabolic sensor, inhibits iTreg differentiation by targeting the Foxp3 protein for degradation [17]. Although HIF-1α is induced by hypoxic conditions, it can also be upregulated through various ways under normoxia [18, 21, 22]. Indeed, our results revealed that cholesterol stimulation stabilized the expression of HIF-1α in CD4^+^ naïve T cells and inhibited their differentiation into iTregs. However, HIF-1a inhibition using a specific inhibitor PX-478, abrogated the suppressive effect of cholesterol and restored the percentage of Foxp3^+^ cells to an even higher level than that in the control group. Recently, PX-478 was reported to ameliorate AS by reducing adipose ceramide production and altering liver lipid metabolism [35, 36]. Our results suggest that PX-478 can act as an immunomodulator that ameliorates AS by promoting iTreg differentiation.

In addition to their damaging effect, mtROS are essential for intracellular redox signaling, where they participate in a wide range of biological processes [37]. Our results showed that mtROS mediated HIF-1a accumulation in iTregs induced by cholesterol stimulation. When mtROS scavenger MT was added to the culture, HIF-1α accumulation was attenuated and the percentage of Foxp3^+^ cells was restored. Furthermore, by utilizing an ODD-Luc reporter Jurkat cell line, we suggested that the accumulation of HIF-1a may be attributed to the mtROS-mediated inactivation of PHDs, enzymes that hydroxylate HIF-1a and target it for proteasomal degradation under normoxia [24].

It was reported that mtROS increased iTreg differentiation by enhancing Foxp3 expression [38], which contradicts our findings. We speculate that mtROS and mtROS-induced HIF-1a may have opposite effects in iTreg differentiation, and the final outcome depends on which of the two plays a stronger role; the nature of the initial stimulus may be crucial in this process. For instance, Liang et al. showed that the natural small molecule piperlongumine enhanced iTreg differentiation by exerting prooxidative effects; however, it also strongly inhibited the expression of HIF-1α [39]. In contrast, our data showed that cholesterol notably increased HIF-1α, which may offset the promotive effect of mtROS in iTreg differentiation.

Regarding nTreg, our main concern was the effect of cholesterol on its immune function. Deregulation of mitochondrial function increased intracellular oxidation and stress, and perturbed immune cell homeostasis and function [27, 40]. mtROS bursts caused by mitochondrial dysfunction could lead to further deterioration of mitochondrial fitness [28]. Futhermore, compared with Tconv cells, human nTregs are more sensitive to oxidative stress [41]. Consistently, we found that cholesterol treatment damaged the structure and function of the mitochondria and induced intracellular oxidative damage in nTregs. However, addition of the mitochondria-targeted ROS scavenger MT to the culture medium successfully restored mitochondrial metabolism, reduced mtROS production, alleviated intracellular oxidative damage, protected the mitochondrial structure, and ultimately improved the immunoregulatory function of nTregs.

Macrophages participate in the entire process of AS through the secretion of pro- or anti-inflammatory cytokines, phagocytosis, removal of necrotic cell debris, and transformation into foam cells. Furthermore, Treg cells can hinder AS progression by affecting the phenotype and function of macrophages. For example, Proto et al. showed that Tregs promoted macrophage efferocytosis by secreting IL-13 [33]. Lin et al. reported that Tregs inhibited macrophage-derived foam cell formation through cell contact and soluble factors [34]. Using an anti-CD25 antibody, Sharma et al. showed that Treg depletion resulted in impaired hallmarks of inflammation resolution in AS, including, but not limited to, alternative activation and efferocytosis of macrophages [9]. However, we found that a high concentration of cholesterol, simulating the microenvironment within AS plaques, impaired the ability of nTregs to license macrophage pro-resolving phenotypes and functions. These results may explain why inflammation in AS plaques is difficult to resolve [4].

Atherogenesis is mostly influenced by the increased production and/or impaired scavenging of ROS in the vascular wall, and mitochondria are one of the main producers of ROS. Targeting mtROS using MT attenuated AS in various ways. For instance, Vendrov et al. showed that MT therapy decreased smooth muscle cell death and matrix degradation in the plaque [42]. Zhu et al. found that clearance of mtROS by MT reversed the promotive effect of ALDH2 knockdown on VSMC senescence [43]. Zeng et al. discovered that MT significantly decreased the production of TNF-α, IL-6 and IL-1β caused by MDM2 overexpression in oxLDL-treated HAECs, which may alleviate AS inflammation [44]. Along with the mechanisms mentioned above, we propose here that MT may act as an immunomodulator to ameliorate inflammation in AS plaques by safeguarding iTreg differentiation and nTreg immunoregulatory function. As presented in Fig. 8, by improving the secretion of multiple cytokines critical to the functional and phenotypic shaping of macrophages, MT successfully rehabilitated the cholesterol-treated nTregs’ ability to license the anti-inflammatory properties of macrophages.

Despite all these benefits, however, it is worth noting that most clinical studies designed to reduce cardiovascular event with antioxidants have failed. One possible reason is that the unstable chemical properties of antioxidants may lead to their premature degradation during delivery and insufficient concentration at the target sites to exert a meaningful therapeutic effect. Nanoparticles (NPs) have displayed great potential in safeguarding active molecules. Compared with the conventional ones, NPs-carried antioxidants, termed nanoantioxidants, have shown the capability of attenuating oxidative stress with better stability and superior effectiveness. What’s more, nanoantioxidants provide the possibility of targeted and temporally controlled delivery of antioxidants at specific body sites, which is especially suitable for AS treatment [45, 46]. Even more interesting is that some NPs showed both ROS-scavenging and anti-inflammatory capacities themselves [47]. We believe that nano-MT may have a better AS-curing effect than common antioxidants and deserve further study.

Finally, it has to be pointed out that cholesterol can also affect Treg through mechanisms other than mitochondria-related ones. Cholesterol metabolism has emerged as a key regulator of T cell response [48]. Our results showed that cholesterol treatment significantly down-regulated *HMGCR* and *HMGCS1* mRNA level in iTreg and nTreg (Additional file 2: Figure S4), suggesting that mevalonate synthesis, the key panel point of cholesterol anabolism, was suppressed by cholesterol stimulation. However, HMGCR inhibition by statin was reported to enhance iTreg differentiation [49], which contradicts with our results. We conjectured that due to the negative feedback inhibition of cholesterol on HMGCR was weaker than statin’s effect, its influence on iTreg polarization may probably be overwhelmed by mtROS-HIF-1a axis. As for nTreg, HMGCR inhibition could also dampen its immunosuppressive activity [50]. Therefore, our results indicated that the mechanism of cholesterol inhibiting nTreg function was not solo one, and besides inducing mitochondrial oxidative stress, cholesterol treatment could also impair nTreg function through down-regulating HMGCR expression. In conclusion, changes in cholesterol metabolism of Treg cells in plaques may affect their differentiation and function, which is worthy of further study.

Several limitations of this study should be considered. First, our study was limited to cell experiments; thus, our results should be validated by animal or clinical studies. Second, HIF-1a increased the differentiation of T helper cell 17 (Th17) while suppressing that of iTregs in mice. However, we did not study the effect of cholesterol on Th17 differentiation because our research mainly focused on Treg. Third, we only studied the effect of soluble cholesterol on Tregs. However, considering the important role of crystalline cholesterol in the pathogenesis of AS, future studies should further explore the effects of crystalline cholesterol on Tregs.

## Conclusions

Our results indicate that cholesterol hinders iTreg differentiation and nTreg immunoregulatory function, which may help explain why inflammation in AS plaques remains difficult to resolve [4]. Furthermore, mtROS mediated the effect of cholesterol on both iTreg and nTreg cells, suggesting that scavenging mtROS may have therapeutic potential in treating AS. Our results highlight the value of lipid-regulating therapy in AS treatment and suggest that MT may be a promising anti-atherogenic drug.

## Supplementary Information

**Additional file 1: Table S1.** Primer sets used in this study. **Table S2.** Gene sequences for lentiviral transfection.

**Additional file 2: Figure S1.** Analysis of nTreg expansion. **Figure S2.** Cholesterol plays a synergistic role with IL-1b in inhibiting iTreg differentiation. **Figure S3.** Statistical analysis of nTreg’s mitochondria. **Figure S4.** Cholesterol treatment inhibited *HMGCR* and *HMGCS1* expression in iTreg and nTreg.

## Data Availability

The datasets used and analyzed during the current study are available from the corresponding author on reasonable request.

## Abbreviations

AS: Atherosclerosis
CTV: CellTrace violet
DMOG: Dimethyloxalylglycine
GFP: Green fluorescent protein
glycoPER: Glycolysis proton efflux rate
HIF-1a: Hypoxia-inducible factor-1a
HREs: Hypoxia-responsive elements
IL-1b: Interleukin 1b
iTregs: Induced Tregs
MLR: Mixed lymphocyte reaction
MMP: Mitochondrial membrane potential
MT: MitoTEMPO
mtROS: Mitochondrial reactive oxygen species
nTregs: Natural Tregs
OCR: Oxygen consumption rate
ODD: Oxygen-dependent degradation domain
oxLDL: Oxidized low-density lipoprotein
Tconv: Conventional T cell
TGF-b: Transforming growth factor-b
Tregs: Regulatory T cells
8-OHdG: 8-Hydroxydeoxyguanosine
