# Supplementary material for: Cholesterol suppresses human iTreg differentiation and nTreg function through mitochondria-related mechanisms
Source: J Transl Med. 2023 Mar 27;21:224. doi: 10.1186/s12967-023-03896-z (PMC10045251; doi:10.1186/s12967-023-03896-z)
Supplement: Supplementary file 1 — Additional file 1: Table S1. Primer sets used in this study. Table S2. Gene sequences for lentiviral transfection. [file 12967_2023_3896_MOESM1_ESM.docx]

**Table S1. Primer sets used in this study**

| *HIF-1α* | GAACGTCGAAAAGAAAAGTCTCG |
| --- | --- |
|  | CCTTATCAAGATGCGAACTCACA |
| *VEGF* | AGGGCAGAATCATCACGAAGT |
|  | AGGGTCTCGATTGGATGGCA |
| *GLUT-1* | GGCCAAGAGTGTGCTAAAGAA |
|  | ACAGCGTTGATGCCAGACAG |
| *CD206* | TCCGGGTGCTGTTCTCCTA |
|  | CCAGTCTGTTTTTGATGGCACT |
| *CD163* | TTTGTCAACTTGAGTCCCTTCAC |
|  | TCCCGCTACACTTGTTTTCAC |
| *CCL22* | ATCGCCTACAGACTGCACTC |
|  | GACGGTAACGGACGTAATCAC |
| *IL10* | GACTTTAAGGGTTACCTGGGTTG |
|  | TCACATGCGCCTTGATGTCTG |
| *Fibronectin* | CGGTGGCTGTCAGTCAAAG |
|  | AAACCTCGGCTTCCTCCATAA |
| *IL6* | CCTGAACCTTCCAAAGATGGC |
|  | TTCACCAGGCAAGTCTCCTCA |
| *IL1β* | ATGATGGCTTATTACAGTGGCAA |
|  | GTCGGAGATTCGTAGCTGGA |
| *TNF-α* | CCTCTCTCTAATCAGCCCTCTG |
|  | GAGGACCTGGGAGTAGATGAG |
| *HMGCR* | TGATTGACCTTTCCAGAGCAAG |
|  | CTAAAATTGCCATTCCACGAGC |
| *HMGCS1* | GATGTGGGAATTGTTGCCCTT |
|  | ATTGTCTCTGTTCCAACTTCCAG |
| *GGPS1* | CGGAAACCACAGCGATTTAGAT |
|  | CCCAAGTGTATTAAGTAGCGGTT |
| *FNTB* | TTTCACCTACTATTGCCCTCCA |
|  | CGTGACTGTTTCCACCGAGT |
| *PGGT1b* | TCTCCGGGCTGGATATGTTG |
|  | CGGAAACCACAGCGATTTAGAT |
| *GAPDH* | ATTCCACCCATGGCAAATTC |
|  | GGATCTCGCTCCTGCAAGATG |
| *18S* | TCAAGAACGAAAGTCGGAGG |
|  | GGACATCTAAGGGCATCACA |

**Table S2. Gene sequences for lentiviral transfection**

| ODD-Luc | CTCGAGGCCACCATGGAATTCAAGTTGGAATTGGTAGAAAAACTTTTTGCTGAAGACACAGAAGCAAAGAACCCATTTTCTACTCAGGACACAGATTTAGACTTGGAGATGTTAGCTCCCTATATCCCAATGGATGATGACTTCCAGTTACGTTCCTTCGATCAGTTGTCACCATTAGAAAGCAGTTCCGCAAGCCCTGAAAGCGCAAGTCCTCAAAGCACAGTTACAGTATTCCAGCAGACTCAAATACAAGAACCTACTGCTAATGCCACCACTACCACTGCCACCACTGATGAATTAAAAACAGTGACAAAAGACCGTATGGAAGACATTAAAATATTGATTGCATCTCCATCTCCTACCCACATACATAAAGAAACTACTGAAGACGCCAAAAACATAAAGAAAGGCCCGGCGCCATTCTATCCGCTGGAAGATGGAACCGCTGGAGAGCAACTGCATAAGGCTATGAAGAGATACGCCCTGGTTCCTGGAACAATTGCTTTTACAGATGCACATATCGAGGTGGACATCACTTACGCTGAGTACTTCGAAATGTCCGTTCGGTTGGCAGAAGCTATGAAACGATATGGGCTGAATACAAATCACAGAATCGTCGTATGCAGTGAAAACTCTCTTCAATTCTTTATGCCGGTGTTGGGCGCGTTATTTATCGGAGTTGCAGTTGCGCCCGCGAACGACATTTATAATGAACGTGAATTGCTCAACAGTATGGGCATTTCGCAGCCTACCGTGGTGTTCGTTTCCAAAAAGGGGTTGCAAAAAATTTTGAACGTGCAAAAAAAGCTCCCAATCATCCAAAAAATTATTATCATGGATTCTAAAACGGATTACCAGGGATTTCAGTCGATGTACACGTTCGTCACATCTCATCTACCTCCCGGTTTTAATGAATACGATTTTGTGCCAGAGTCCTTCGATAGGGACAAGACAATTGCACTGATCATGAACTCCTCTGGATCTACTGGTCTGCCTAAAGGTGTCGCTCTGCCTCATAGAACTGCCTGCGTGAGATTCTCGCATGCCAGAGATCCTATTTTTGGCAATCAAATCATTCCGGATACTGCGATTTTAAGTGTTGTTCCATTCCATCACGGTTTTGGAATGTTTACTACACTCGGATATTTGATATGTGGATTTCGAGTCGTCTTAATGTATAGATTTGAAGAAGAGCTGTTTCTGAGGAGCCTTCAGGATTACAAGATTCAAAGTGCGCTGCTGGTGCCAACCCTATTCTCCTTCTTCGCCAAAAGCACTCTGATTGACAAATACGATTTATCTAATTTACACGAAATTGCTTCTGGTGGCGCTCCCCTCTCTAAGGAAGTCGGGGAAGCGGTTGCCAAGAGGTTCCATCTGCCAGGTATCAGGCAAGGATATGGGCTCACTGAGACTACATCAGCTATTCTGATTACACCCGAGGGGGATGATAAACCGGGCGCGGTCGGTAAAGTTGTTCCATTTTTTGAAGCGAAGGTTGTGGATCTGGATACCGGGAAAACGCTGGGCGTTAATCAAAGAGGCGAACTGTGTGTGAGAGGTCCTATGATTATGTCCGGTTATGTAAACAATCCGGAAGCGACCAACGCCTTGATTGACAAGGATGGATGGCTACATTCTGGAGACATAGCTTACTGGGACGAAGACGAACACTTCTTCATCGTTGACCGCCTGAAGTCTCTGATTAAGTACAAAGGCTATCAGGTGGCTCCCGCTGAATTGGAATCCATCTTGCTCCAACACCCCAACATCTTCGACGCAGGTGTCGCAGGTCTTCCCGACGATGACGCCGGTGAACTTCCCGCCGCCGTTGTTGTTTTGGAGCACGGAAAGACGATGACGGAAAAAGAGATCGTGGATTACGTCGCCAGTCAAGTAACAACCGCGAAAAAGTTGCGCGGAGGAGTTGTGTTTGTGGACGAAGTACCGAAAGGTCTTACCGGAAAACTCGACGCAAGAAAAATCAGAGAGATCCTCATAAAGGCCAAGAAGGGCGGAAAGATCGCCGTGTAAGGATCC |
| --- | --- |
| HREs-GFP | GGATCCTACGTGCTGTTACGTGCTGTGTACGTGCTGTTACGTGCTGTGTACGTGCTGTTACGTGCTGTAAGCTTAGACACTAGAGGGTATATAATGGAAGCTCGACTTCCAGCTTGGCAATCCGGTACTGTTGGTAAAGCCACCATGGTGAGCAAGGGCGAGGAGCTGTTCACCGGGGTGGTGCCCATCCTGGTCGAGCTGGACGGCGACGTAAACGGCCACAAGTTCAGCGTGTCCGGCGAGGGCGAGGGCGATGCCACCTACGGCAAGCTGACCCTGAAGTTCATCTGCACCACCGGCAAGCTGCCCGTGCCCTGGCCCACCCTCGTGACCACCCTGACCTACGGCGTGCAGTGCTTCAGCCGCTACCCCGACCACATGAAGCAGCACGACTTCTTCAAGTCCGCCATGCCCGAAGGCTACGTCCAGGAGCGCACCATCTTCTTCAAGGACGACGGCAACTACAAGACCCGCGCCGAGGTGAAGTTCGAGGGCGACACCCTGGTGAACCGCATCGAGCTGAAGGGCATCGACTTCAAGGAGGACGGCAACATCCTGGGGCACAAGCTGGAGTACAACTACAACAGCCACAACGTCTATATCATGGCCGACAAGCAGAAGAACGGCATCAAGGTGAACTTCAAGATCCGCCACAACATCGAGGACGGCAGCGTGCAGCTCGCCGACCACTACCAGCAGAACACCCCCATCGGCGACGGCCCCGTGCTGCTGCCCGACAACCACTACCTGAGCACCCAGTCCGCCCTGAGCAAAGACCCCAACGAGAAGCGCGATCACATGGTCCTGCTGGAGTTCGTGACCGCCGCCGGGATCACTCTCGGCATGGACGAGCTGTACAAGTAACTCGAG |
