# Supplementary material for: Cholesterol suppresses human iTreg differentiation and nTreg function through mitochondria-related mechanisms
Source: J Transl Med. 2023 Mar 27;21:224. doi: 10.1186/s12967-023-03896-z (PMC10045251; doi:10.1186/s12967-023-03896-z)
Supplement: Supplementary file 2 — Additional file 2: Figure S1. Analysis of nTreg expansion. Figure S2. Cholesterol plays a synergistic role with IL-1b in inhibiting iTreg differentiation. Figure S3. Statistical analysis of nTreg’s mitochondria. Figure S4. Cholesterol treatment inhibited HMGCR and HMGCS1 expression in iTreg and nTreg. [file 12967_2023_3896_MOESM2_ESM.docx]

**
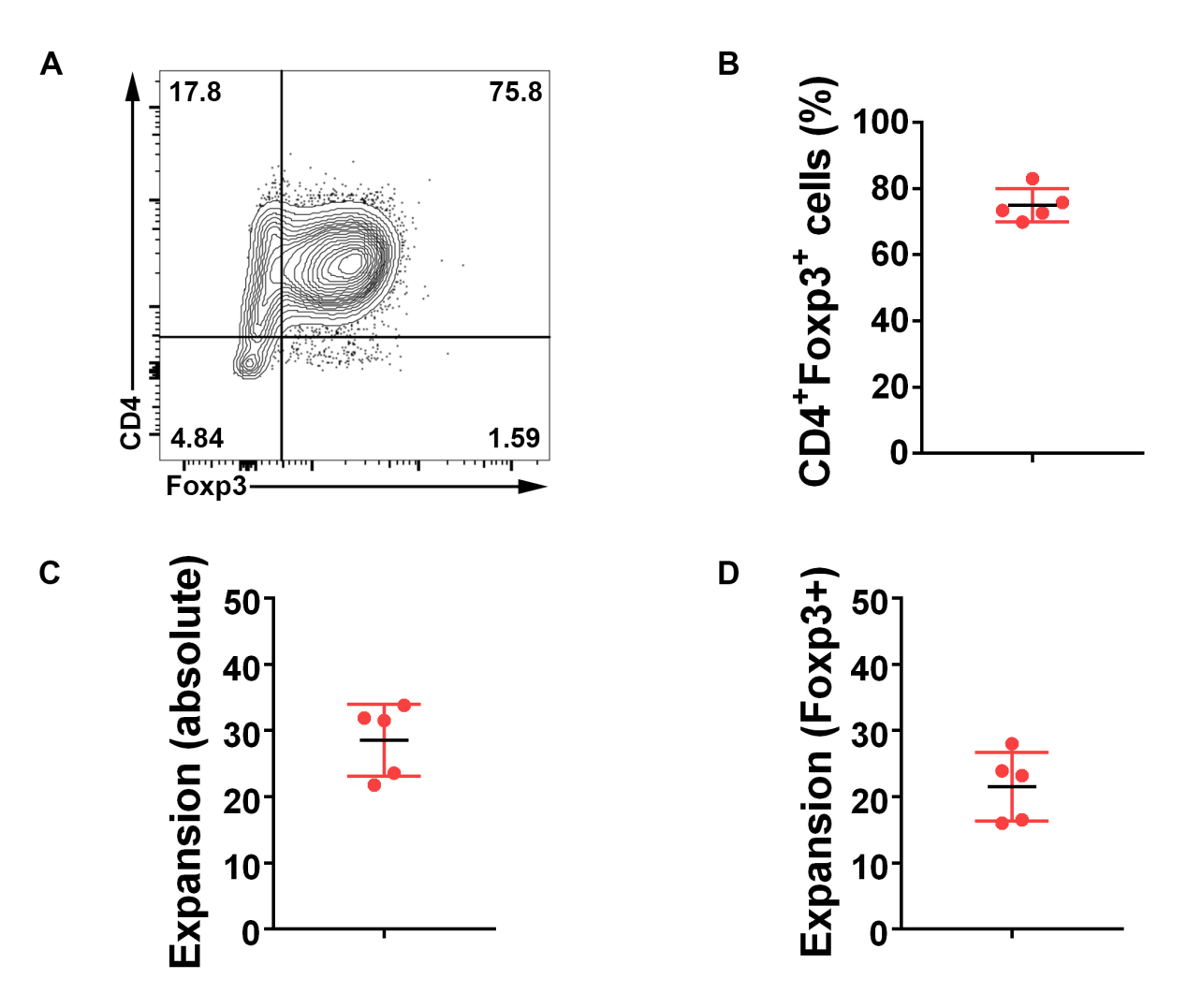
**

**Figure S1. Analysis of nTreg expansion.** MACS isolated human CD4^+^CD25^+^CD127^dim/-^ nTreg cells were expanded for 14 days in vitro. Afterwards, cells were fluorescently stained with FITC-CD4 and PE-Foxp3 antibodies, and analyzed with flow cytometry. **A** Representative flow cytometric plot of CD4^+^Foxp3^+^ nTreg cells. **B** Frequency of Foxp3^+^ cells after expansion from 5 experiments. **C, D** Fold expansion of total cells (C) and FoxP3+ cells (D). Each dot represents one experiment.

**
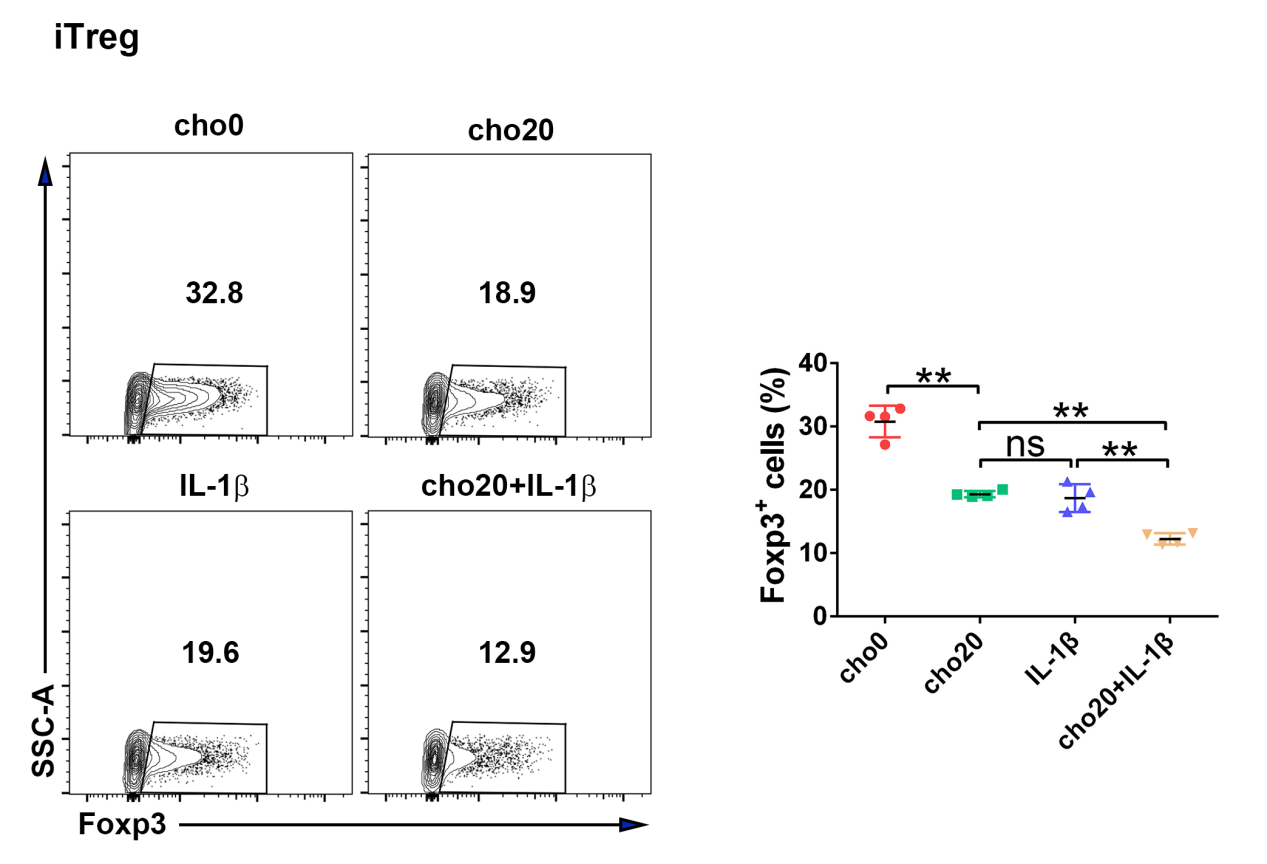
**

**Figure S2. Cholesterol plays a synergistic role with IL-1β in inhibiting iTreg differentiation.** Naïve T cells were polarized for 2 days *in vitro* with cholesterol added in the culture alone or combined with IL-1β. Frequency of Foxp3^+^ cells was analyzed by flow cytometry. n=4 per group. Data are expressed as the mean ± SEM. **P*＜0.05, ***P*＜0.01.


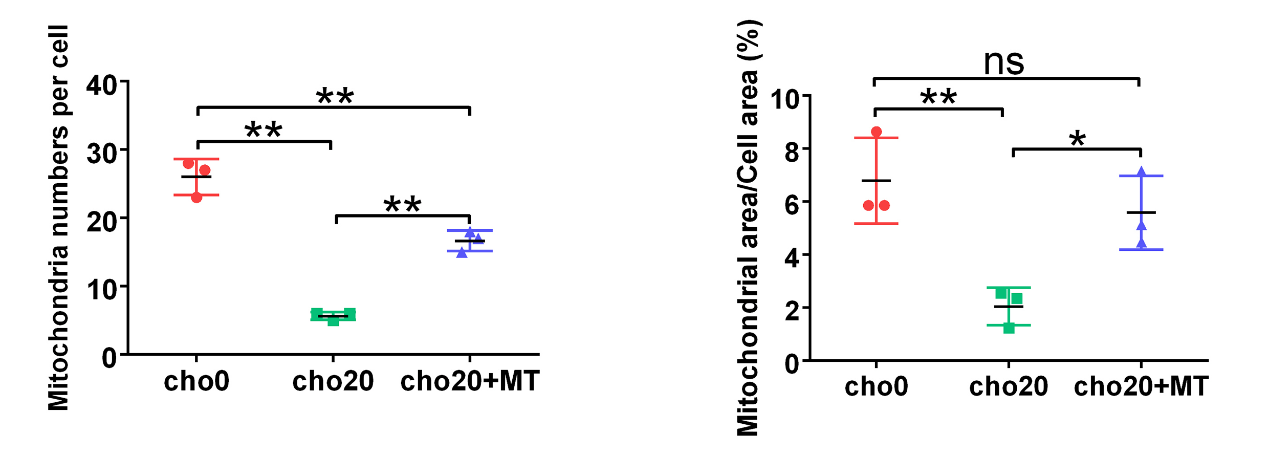


**Figure S3. Statistical analysis of nTreg’s mitochondria.** Mitochondria numbers per cell and mitochondrial area/cell area was counted and calculated. n=3 per group. Data are expressed as the mean ± SEM. **P*＜0.05, ***P*＜0.01.


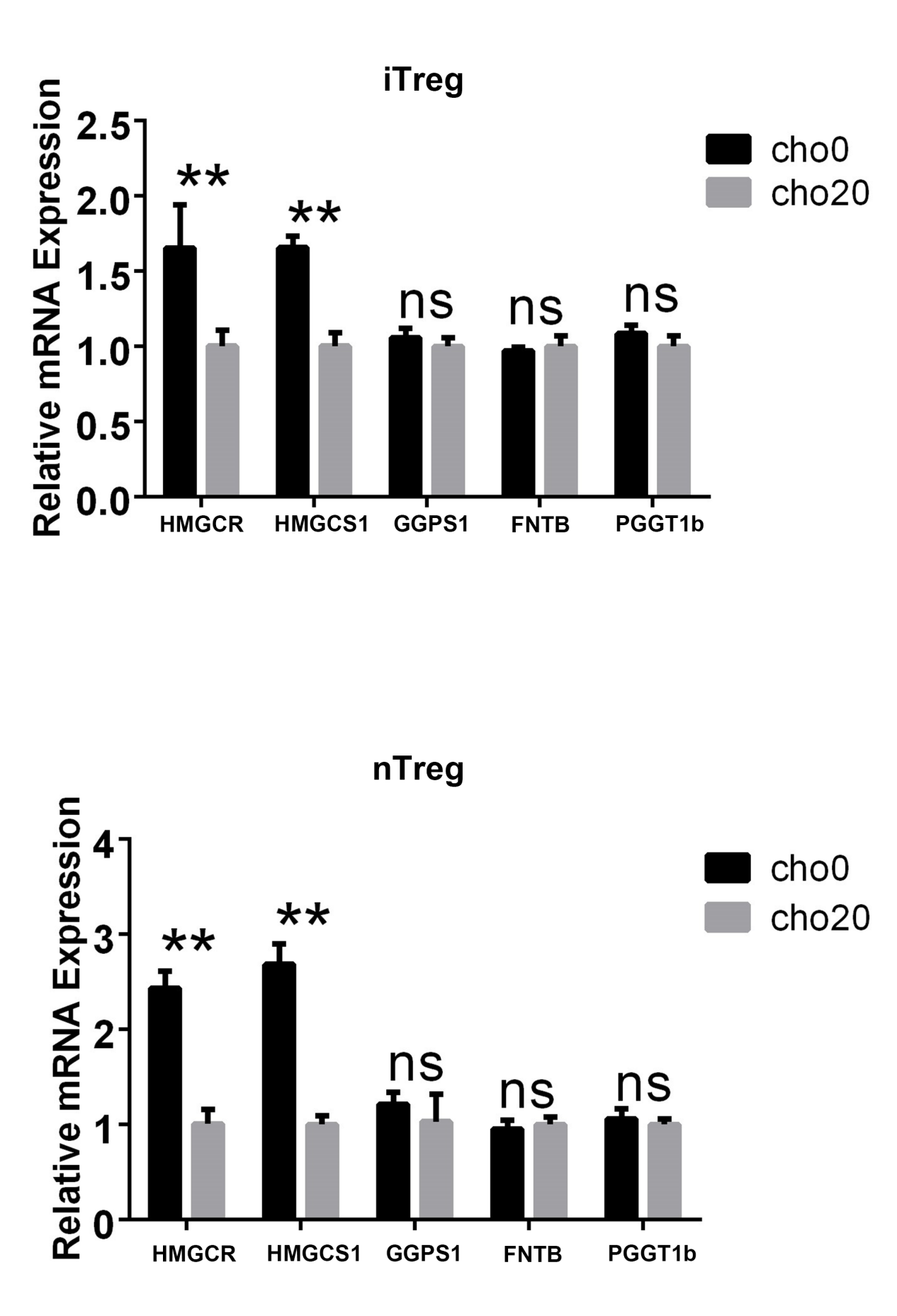


**Figure S4. Cholesterol treatment inhibited *HMGCR* and *HMGCS1* expression in iTreg and nTreg.** A. Naïve T cells were polarized into iTreg for 2 days with or without cholesterol added in the culture. B. *In vitro* expanded nTregs were treated with cholesterol for 16 h. Relative mRNA levels were analyzed by RT-qPCR. n=4 per group. Data are expressed as the mean ± SEM. **P*＜0.05, ***P*＜0.01.
